# Closed Compression Nailing Using a New-Generation Intramedullary Nail without Autologous Bone Grafting for Humeral Shaft Nonunion

**DOI:** 10.1155/2021/5548729

**Published:** 2021-04-11

**Authors:** Genta Fukumoto, Tomoaki Fukui, Keisuke Oe, Atsuyuki Inui, Yutaka Mifune, Ryosuke Kuroda, Takahiro Niikura

**Affiliations:** Department of Orthopaedic Surgery, Kobe University Graduate School of Medicine, 7-5-1 Kusunoki-cho, Chuo-ku, Kobe 650-0017, Japan

## Abstract

**Introduction:**

Although the recommended treatment for humeral shaft nonunion is compression plating with autologous bone grafting, we treated a case of humeral shaft nonunion with an intramedullary nail (IMN) without bone grafting. *Presentation of Case*. Osteosynthesis with IMN was performed on a 24-year-old man with a humeral shaft fracture at another hospital. However, bony union was not obtained 1 year after the first surgery, and he was referred to our institution. We treated the nonunion with exchange nailing without autologous bone grafting using compression function of the nail, leading to bony union at 7 months postoperatively. At the final follow-up 2 years and 4 months postoperatively, the patient had full range of motion in the left shoulder and elbow joints. *Discussion*. Compression plating with autologous bone grafting is reported to be the gold standard for the treatment of humeral shaft nonunion. IMN is advantageous for minimal invasion; however, the conventional type of IMN cannot apply compression force between fragments and does not have sufficient stability against rotational force. In this case, we used an IMN that could apply compression between the fragments and which had rotational stability via many screws. We did not perform bone grafting because the current nonunion was adjudged to be biologically active, and we achieved good functional results.

**Conclusion:**

We treated humeral shaft nonunion using IMN with compression, but without bone grafting, leading to successful clinical outcomes. This strategy might be an appropriate choice for the treatment of humeral shaft nonunion with biological activity.

## 1. Introduction

Humeral shaft fractures are common and account for 3–5% of all fractures [1, 2]. Generally, fractures can be treated conservatively or surgically, unless open fractures, polytrauma, or progressive radial nerve deficits are involved [3–5]. Nonunion following humeral shaft fractures has been reported to occur in 0.3–13% of cases after conservative or surgical treatment [6, 7]. Nonunion of the humeral shaft can be treated with compression plates, intramedullary nail (IMN), and external fixation. The generally recommended method is compression plating with autologous bone grafting [8]. However, we treated a case of humeral shaft nonunion using IMN without autologous bone grafting by considering the cause of the nonunion, and bony union was achieved.

## 2. Presentation of Case

**Figure 1:**
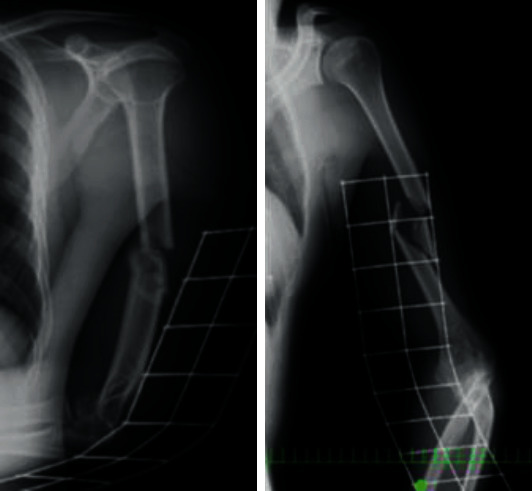
Radiographs of a left humeral shaft fracture. A fracture can be seen with the third and fourth fragments in the mid shaft. The AO-OTA classification of this fracture is 12-C3.

**Figure 2:**
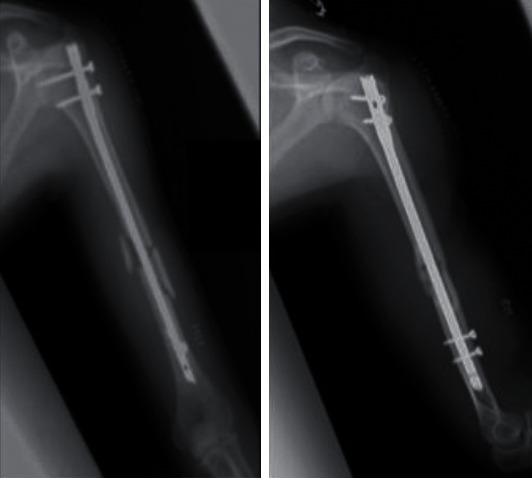
Radiographs just after the first surgery using IMN. The surgery was performed using a curved nail 8 days after the injury.

**Figure 3:**
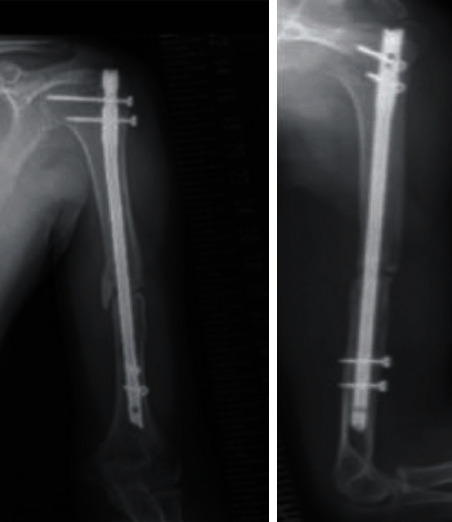
Radiographs at the first visit to our hospital. Nonunion was observed with callus formation on the medial side.

**Figure 4:**
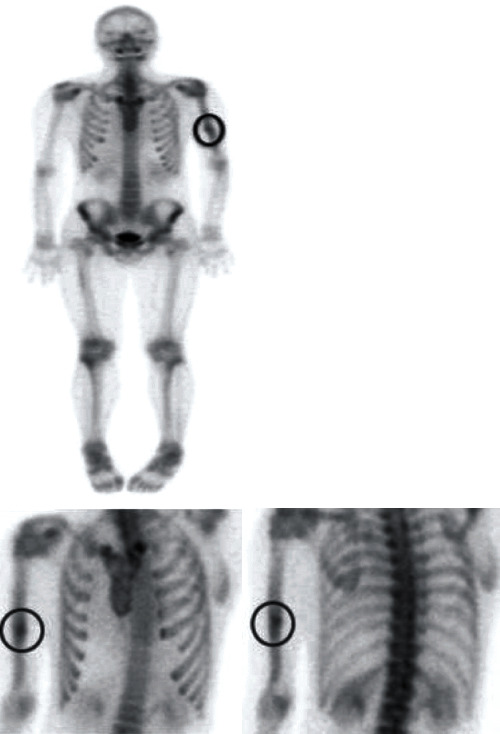
Bone scintigraphy showing intense accumulation at the nonunion site of the left humerus. The black circles indicate the nonunion site.

**Figure 5:**
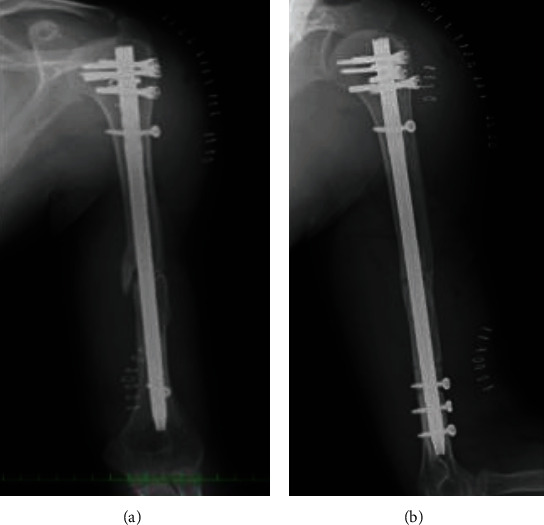
Radiographs just after the nonunion surgery was performed at our hospital. The gap between bone fragments seemed to be reduced by compression ((a) before nonunion surgery and (b) after the nonunion surgery). The compression screw appeared to be curved.

**Figure 6:**
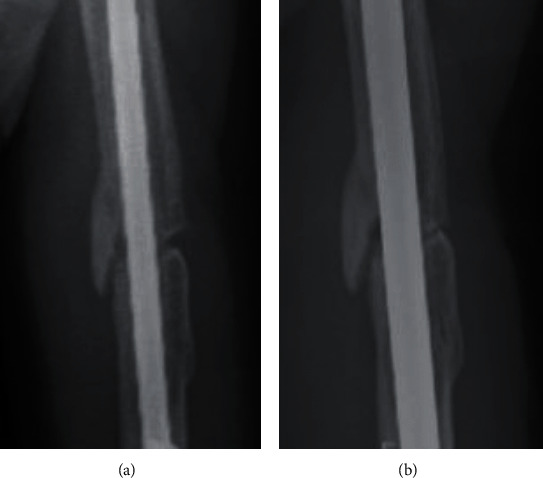
Radiographs comparing the gap before and after the nonunion surgery. (a) The gap before the surgery and (b) the gap after the surgery. It can be noted that the gap was reduced by compression.

**Figure 7:**
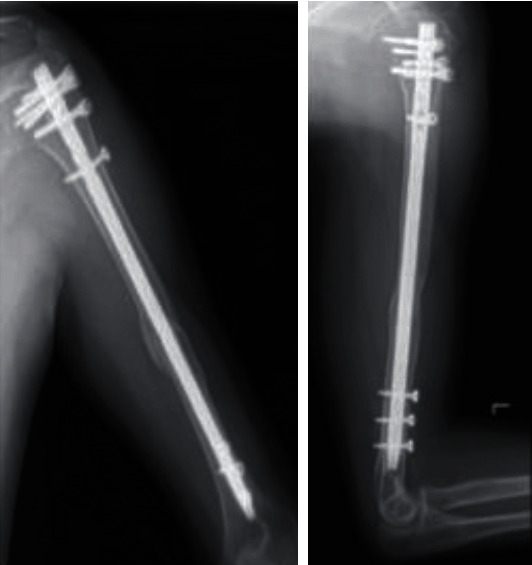
Radiographs at 7 months after the surgery. Bony union was obtained. At 15 months after the second surgery, we performed surgery to remove all implants including the screws and the nail.

**Figure 8:**
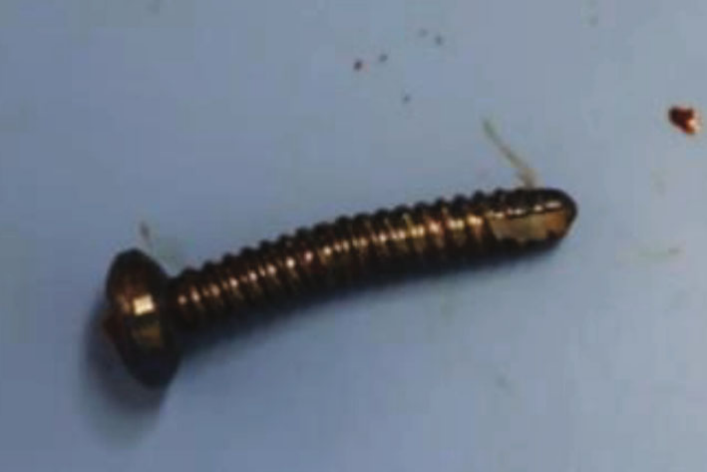
The compression screw removed after bone union was bent because of the compression force.

**Figure 9:**
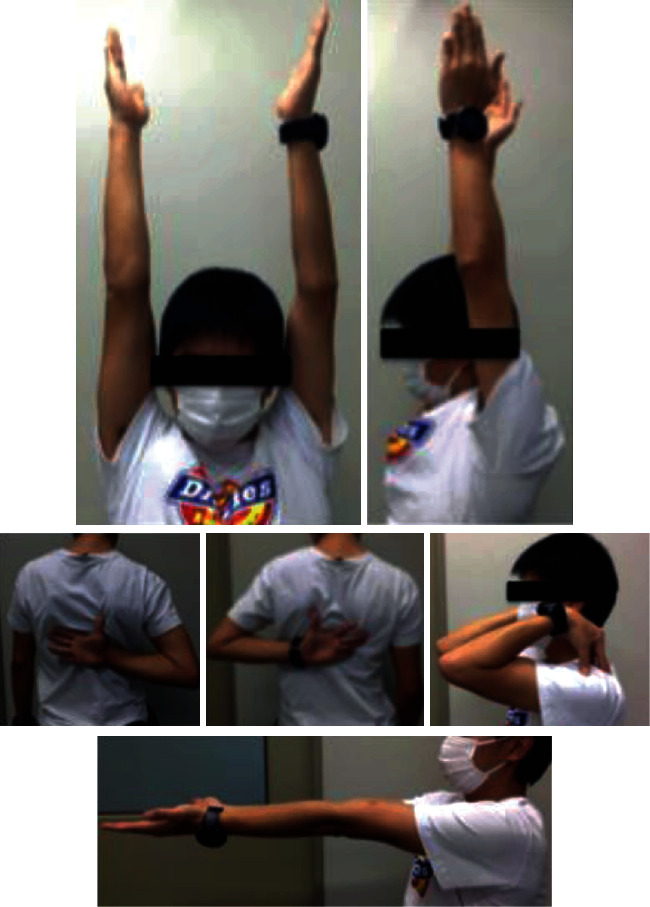
Range of motion of the left shoulder and elbow joints was not limited at the final follow-up 2 years and 4 months postoperatively.

A 24-year-old man injured his upper limb during snowboarding and was diagnosed with a left humeral shaft fracture, which was categorized as 12-C3 based on the AO-OTA classification (Figure 1). Osteosynthesis with IMN (diameter 7 mm) was performed at another hospital (Figure 2). Four months after the procedure, the patient started low-intensity pulsed ultrasound therapy to facilitate bony union. However, 10 months after the procedure, the patient still experienced pain in his left upper arm, and bony union was not obtained. Therefore, he was referred to our hospital for further treatment. On radiography, callus formation was observed (Figure 3), and bone scintigraphy showed high uptake at the nonunion site (Figure 4). Since these findings suggest that biological activity is preserved in the nonunion site, we considered that the cause of nonunion would be insufficient stability because of the thin diameter of the inserted nail and the few screws used. Therefore, we performed exchange nailing and used the MultiLoc® Humeral Long Nail (DePuy Synthes, West Chester, USA), which is a thicker nail (diameter 8.5 mm) that uses more locking screws, including 5 proximal screws with 3 screw-in-screws and 3 distal screws (Figure 5). Furthermore, during the surgery, compression was applied between the bone fragments using compression function, in which the compression screw inserted in the proximal fragment was pushed down to the distal fragment by approximately 4 mm. The gap between the bone fragments was reduced (Figure 6). Using an image intensifier, we noted that the compression screw was bent due to the compression. We did not open the nonunion site or perform autologous bone grafting. After immobilization with a triangular bandage for 1 week, passive and active range of motion training of the left shoulder was started as postoperative therapy. Seven months after the operation, we confirmed bony union on radiography (Figure 7), and the patient had good functional recovery. Fifteen months after the procedure, we removed the implants. When we removed the implants, we confirmed that the screw was bent (Figure 8). At the final follow-up 2 years and 4 months after the nonunion surgery, the patient had full range of motion in the left shoulder and elbow joints (Figure 9) and did not experience any pain. Before nonunion surgery and at the final follow-up, the quality of life (QOL) was assessed using the Short Form- (SF-) 36 health survey, which is a validated and generally accepted instrument [9]; upper limb function was similarly assessed with the Disabilities of the Arm, Shoulder, and Hand Questionnaire (DASH) score [10]. The SF-36 is divided into 8 domains with a national standard value of 50 and with greater values indicating a better status. The DASH score ranges between 0 and 100, with lesser values indicating better function. These assessments revealed that the patient's QOL and upper limb function had notably improved at the final follow-up compared with the prenonunion surgery status (Table 1).

## 3. Discussion

With regard to nonunion rates following humeral shaft fracture, some studies have reported the lack of a difference in nonunion rates between surgical and nonsurgical treatments, while some have reported lower nonunion rates in nonsurgical treatments than in surgical treatments [7, 11, 12]. These studies may have contained a selection bias, that is, simple fractures tend to be managed conservatively and more complicated fractures, operatively. The causes of humeral shaft nonunion include infection, distraction at the fracture site, soft tissue interposition, unstable fixation, and wrong choice of implant [2]. In terms of factors that inhibit fracture healing, smoking, diabetes, medications, malnutrition, and noncompliance with physicians' instructions have been implicated [13].

Various devices are used in the surgical treatment of humeral shaft nonunion: compression plates, IMN, external fixators, and bone graft struts [14, 15]. Peters et al. [8] reviewed 36 papers on the treatment of humeral shaft nonunion and reported a bony union rate of 98% and complication rate of 19% in plate fixation with autologous bone grafting (*n* = 672). The bony union rate was 66%, and the complication rate was 1% in IMN without autologous bone grafting (*n* = 78). They concluded that plate fixation with autologous bone grafting is the best treatment for humeral shaft nonunion, and they did not recommend IMN because of the lower union rates. It has been suggested that IMN has lower bone union rates because of a lack of compression force on the fracture site and poor rotational stability [2, 16]. Augmentative plating with nail retention also can be a choice of treatment for humeral shaft nonunion. Gessman et al. [17] reported good treatment outcomes for humeral shaft nonunion by anterior augmentative plating. They reported high union rates as 97% (*n* = 37). In addition, Allende et al. [18] reported humeral shaft nonunions achieved bony union using minimally invasive plate osteosynthesis and there were no infections or postoperative nerve disorder. However, Kesemenli et al. reported that IMN is a suitable choice for treating humeral shaft nonunion because of its low rate of infection, low risk of injury to the radial nerve, and low requirement for soft tissue dissection [19].

Generally, several factors are required for fracture healing. Giannoudis et al. included these important factors, such as osteogenic cells, osteoconductive scaffolds, osteoinductive growth factors, and mechanical environment, in the “diamond concept” [20]. These factors are usually divided into biological factors, which include the first three factors, and mechanical factors. When treating nonunion, it is important to determine the causative factor. Atrophic nonunion is usually associated with a lack of biological factors at the fracture site [21]. The mainstay of surgical treatment for nonunion with low biological activity is autologous bone grafting [22]. However, hypertrophic nonunion is usually associated with insufficient stability; therefore, surgical intervention for this type of nonunion must be aimed at improving stability [23]. Gunesh et al. reported that 26% of patients with bone grafts harvested from the iliac crest experienced pain and limited mobility at the donor site, and 13% had discomforting paresthesia around the thigh of the donor side 1 year after the surgery [24]. Considering these, unnecessary autologous bone collection should be strictly avoided.

In the current case, a little callus and a slight bony gap in the nonunion site were recognized on radiography, but it was difficult to judge whether biological activity was preserved via radiography alone. On bone scintigraphy, obvious accumulation was observed at the nonunion site, indicating that the biological activity was sufficient. Based on these findings, we considered that the nonunion was not due to decreased biological activity but due to an inappropriate mechanical environment. In other words, improvement of stability was necessary to achieve bony union. Therefore, we performed exchange nailing without autologous bone grafting, leading to bony union with sufficient QOL and upper limb function. One of the features of this implant is a compression system that can apply compression force between the bone fragments by pushing down the compression screw inserted in the proximal fragment. Another feature is that more screws can be inserted into this device compared to the conventional IMN. This device uses more locking screws, comprising 5 proximal screws with 3 screw-in-screws and 3 distal screws. Sufficient rotational stability of the bone fragments was expected because of these screws. The current treatment indicates that the IMN without autologous bone grafting might be the best method for the treatment of humeral shaft nonunion by identifying the cause of the nonunion, understanding the implant characteristics, and using this information appropriately.

## 4. Conclusions

It is important to consider the causative factors when treating nonunion. We performed exchange nailing without autologous bone grafting to treat humeral shaft nonunion based on the suspected cause of the nonunion and obtained bony union and good functional outcomes. It might be an innovative choice to treat nonunion using only IMN. However, exchange nailing applying compression between fragments via proper implants, without autologous bone grafting, might be an appropriate method for treating humeral shaft nonunion with biological activity.

## Figures and Tables

**Table 1:** The SF-36 score and DASH score at the prenonunion surgery and final follow-up. Higher SF-36 and lower DASH scores indicate better status and function. The QOL and the upper limb function were notably improved at the final follow-up compared with the prenonunion surgery status.

		Prenonunion surgery	Final follow-up (postop 2 years and 4 months)
SF-36	Physical functioning	50.6	57.8
	Physical role functioning	42.4	55.7
	Bodily pain	39.8	61.7
	General health perceptions	54.8	60.2
	Vitality	49.8	53.0
	Social role functioning	57.0	57.0
	Emotional role functioning	56.1	56.1
	Mental health	51.8	59.9
DASH score		35	0

## Data Availability

The data used to support the findings of this study are included within the article.
